# Of mice and gender: considering gender norms in rodent models

**DOI:** 10.1186/s13293-026-00909-6

**Published:** 2026-05-30

**Authors:** Liv Engel, Gillian Einstein, Robert R. Rozeske

**Affiliations:** 1 Department of Psychology, University of Toronto – Scarborough, Toronto, ON Canada; 2 Department of Psychology, University of Toronto, Toronto, ON Canada; 3 Department of Cell and Systems Biology, University of Toronto, Toronto, ON Canada; 4 Scientific Associate, Baycrest Hospital, Toronto, ON Canada

**Keywords:** Sex, Gender, Rodents, Gender Norms, Roles, context, environment

## Abstract

**Background:**

Sex and gender are often used interchangeably, though they refer to distinct concepts —the former relating to biological differences grounded in chromosomal and hormonal features and the latter to socially constructed norms, roles, and identity. In preclinical research with non-human animals, the prevailing view is that only sex differences are measurable because it is commonly accepted that rodents do not express a gender identity. However, we argue that rodents express context-specific, sex-specific behaviors which fulfill the defining requirement of *gender-like norms*. To explore whether sex-specific behavioral patterns in non-human animals provide evidence of gender-like norms, we focus on rodents, the most common non-human animal model. We cite examples of how their sex-specific behavior is shaped by their environment. Importantly we acknowledge that gender norms are malleable depending on the physical and social contexts – a dynamic mix of spatial environment, material composition, and social structure that jointly shapes how behavior unfolds and its meaning. Thus, our analysis examines these gender-like norms across three levels. First, we compare natural and vivarium environments to reveal the malleability of gender-like norms. We then explore how the material composition in the physical context of the vivarium cage (e.g., cage type, enrichment) can shift these norms. Finally, we show the social context within the cage (e.g., sex composition, rank, familiarity) modifies individual interactions which in turn, affects the expression of gender-like norms. Throughout we provide experimental design recommendations that advocate for the explicit consideration of both sex and gendered behavior in rodent models.

**Conclusion:**

Incorporating these gender considerations will yield more effective and translatable animal models, significantly improving our ability to discover and test treatments for human toxicology, physiology, psychopathology, and neurological diseases.

## Highlights

Rodents are often assumed not to have gender, yet they exhibit sex-specific, context dependent, roles that can reflect aspects of human gender norms.

A vivarium’s physical and social contexts can shape sex-specific roles to form rodent gender-like norms.

Rodent gender-like norms are often overlooked in research because the physical and social contexts that construct them are underreported, thus resulting in poorer translational animal models for the human condition.

Reporting factors such as cage type, housing density, enrichment type, and interactions with enrichment and conspecifics in the cage environment, and performing sex-based analyses on these factors, can clarify gender-like behavior in rodents and improve the reproducibility and translatability of preclinical rodent research.

## Background

In this commentary, we argue that the foundation of gender norms is how physical and social contexts define the specific roles and responsibilities according to sex. Incorporating these considerations into preclinical rodent models could lead to more meaningful research questions and methodologies, enhancing our understanding of individual factors that influence behavior as well as biology facilitating the development of effective personalized treatments for humans. It is imperative to develop specific preclinical research models that can facilitate studying the effects of social norms related to sex or what we call, gender-like norms, for both reasons of translation from animal models to humans and reproducibility of rodent experiments. Approximately 90% of drugs that are effective in animal models fail in clinical trials, amounting to an estimated $28 billion per year in the United States spent on preclinical studies that cannot be reproduced [1, 2]. In addition, rodent behavioral findings vary from lab-to-lab, even when strain and sex are controlled for, suggesting there may be other factors modulating these behaviors [3, 4]. We cannot isolate a single reason for this failure or variation, but we propose that consideration of how physical and social contexts interact with sex-specific roles to produce gender-like norms defined here as the consistent, context-dependent patterns of sexed behavior will provide a necessary framework to enrich our explanatory power and ultimately improve reproducibility.

A framework that considers sex-specific roles in rodents requires preclinical researchers to examine behavioral expression of gender-like norms. There is pushback to the idea that rodents have any type of gender since gender is often taken to only be gender identity [5–7] or *one’s view of themselves* or the *gender identification of the individual* [8]. Indeed, we cannot ask a rodent to articulate their gender identity, and it is unclear if such a concept is meaningful to them. Thus, here we are not proposing a metaphor for gender identity but for gender-like norms [8] or *the set of responsibilities*,* functions*,* or roles of an individual that are highly dependent on the cultural context* [8]. We use norm in the sense of something that is usual, typical, or standard. Therefore, a gender norms framework critically depends on culture. The observations of social learning in preclinical research settings are consistent with rodents meeting the minimal conditions for culture. For example, transmission of food preference is an ecologically-relevant behavior that is socially learned, shared within a group, and can persist across time [9]. In rodent preclinical research, we define culture as the interaction of physical and social contexts that guide behavior. With these operational definitions in place, the study of gender norms in rodents becomes a tractable research question. Focusing on observable, context-dependent behaviors, rather than unreachable internal identities, allows rodent researchers to model gender-like complexities of human health. We contend that a gender-like norms framework allows sex-specific roles into rodent models for human health that classic context-dependent behavior alone cannot explain.

Current frameworks that treat ‘sex’ and ‘context’ as independent variables fail to account for the stable, sex-specific roles that emerge from their interaction. The interaction of sex-specific roles and context in rodent research is a phenomenon unto itself that we define as “gender-like norms”. We use gender-like norms as a metaphor for sex-specific roles and expectations that exist in human societies. Metaphors for what we think of as distinctly human attributes in animal models are common and have produced invaluable translatable insights. For example, Eric Kandel successfully used conditioned reflexes in sea slugs as an experimental metaphor of ‘memory’, launching the study of synaptic plasticity [10] without claiming that sea slugs possess the consciousness that accompanies human memory. In this example, ‘memory’ describes the functional outcome of neuroplasticity. In the gender-like norms framework, ‘gender-like’ describes the functional outcome of sex-lab culture interactions. Therefore, our framework is not merely a rebranding of contextual modulation. Instead, it provides a preclinical model more closely aligned to the human condition as well as the explanatory power to address the reproducibility crises within rodent labs [2, 3, 11]. For example, a gender-like norms framework can identify how disparate lab ‘cultures’ (i.e. unique combinations of physical and social contexts) produce different, yet internally consistent, behaviors [3]. The metaphor of gender-like norms may help clarify why some sex-based findings fail to replicate across labs such as in the example of outcome devaluation where some labs find females are more habitual in their responding [12, 13], some find males are [14], and some find no sex difference [15, 16].

The essential first step to use a gender-like norms framework in rodents is to provide operational definitions. Below we define the terms we use throughout the paper.

- *Sex-specific role*: Patterned, baseline behaviors stereotypically associated with either being male (e.g. ‘males are aggressive’) or female (e.g. ‘females are maternal’).
- *Physical context*: The tangible, non-living, attributes of the environment (architecture, materials, space).
- *Social context*: The dynamic interactions and relationships between rodents that occur within the physical context.
- *Lab culture*: The unique physical and social context within a lab environment.
- *Gender-like behavior*: The result of a sex-specific role that is filtered through physical and social contexts. For example, while ‘aggression’ may be a sex-specific role for males, the expression of that behavior could be considered gender-like since it depends on the cage architecture and the hierarchy of the colony [17]. In females, maternal behavior can be viewed as a sex-specific behavior, but again, this is a role that can change depending on the presence of conspecifics and the physical context.
- *Gender-like norms*: A set of context-dependent, sex-specific roles that are predictable for a population within a specific lab culture. Analogous with humans [18], this distinction between gender-like norms and sex-specific roles allows us to view sex as a fixed variable that dynamically interacts with the social and physical context to produce unique outcomes. Ultimately, ignoring gender-like norms in rodent models can confound the accurate interpretation of experimental results and can reduce their translational relevance to model complex human behaviors.

Gender-like norms are not simply sex-specific roles; to operationally distinguish the two we propose a framework based on context dependency. We define sex-specific roles as traits or behaviors that remain relatively invariant across diverse physical and social contexts (e.g., basic reproductive behavior). In contrast, we define gender-like norms as sex-specific roles that are significant in one context but are modified, abolished, or reversed in another [19–21]. This distinction is empirically testable by observing if a sex difference in behavior (such as risk-taking or aggression) fluctuates based on aspects of the context such as cage enrichment or social hierarchy. If the sex difference is modified by the lab culture, it indicates that that behavior is a gender-like norm rather than an immutable sex-specific trait. For example, female rats displaying aggression when males are absent from the environment suggests this trait is modified by context [19]. By adopting this framework, researchers can move beyond this analogy to human gender norms, which depend on cultural context. In this way, researchers can model gender-like norms and thus, their effects on biology, in rodent models.

Here we discuss examples of rodent gender-like norms (i.e., context-specific, sex-specific roles), arguing that their biology is shaped by physical and social contexts that can reflect aspects of human gender norms.

We explore this across three levels relevant to preclinical rodent models:

1. Nature and Vivarium: A comparison of the natural environment with that of the vivarium describing how these disparate physical and social contexts produce unique sets of gender-like norms.
2. Physical Context: An exploration of the cage within the vivarium, demonstrating how factors like cage type and enrichment shift gender-like norms.
3. Social Context: A discussion of the individual interactions within the cage, illustrating how the presence of conspecifics modifies the expression of gender-like norms.

By laying out the conditions under which sex-specific roles vary, we advocate for a complex understanding of rodent gender-like behavior that considers a rodent’s individual housing conditions and global lab environment to provide specific recommendations to improve current research practices and enhance the translational value of models for human toxicology, physiology, psychopathology and neurological diseases.

## Nature and vivarium: two environments that create unique sets of gender-like norms

The comparison of natural and vivarium environments offers a key opportunity to examine how their distinct physical and social contexts produce unique gender-like norms. In natural environments, rodents live in larger colonies often with limited access to resources. In contrast, in a vivarium environment, rodents typically live in smaller colonies with unlimited access to resources. Drawing from research on rodents in natural environments, each animal’s role varies depending on the size and proximity to resources of the colony [22]. This parallels how human gender roles vary across societies with differing expectations [23–25]. Here, we explore how these factors of colony size and access to resources in the natural environment and the vivarium’s caged environments construct distinct physical and social contexts that produce distinct sex-specific expectations, or more simply, gender-like norms.

### Colony size

Wild rats typically live in colonies of around 10–15 individuals, where male rats form stable social hierarchies [22]. Wild mice, in contrast to rats, tend to not live in complex social environments and are more individualistic, but when placed in confined group settings, male mice establish social hierarchies [26–28]. Unlike rats, who form stable dominance hierarchies, male mice in confined settings engage in constant rivalries [29]. Typically, in a vivarium, rodents are housed in a cage environment with 2–4 of their litter mates. This physical context in male mice can create competition for dominance [30] that shapes the social dominance hierarchies. Dominance competition creates a more stressful experience for male mice compared to females, who do not establish dominance hierarchies. The stress produced by social dominance in males can alter other behaviors, brain circuitry, and neurotransmitter release [31]. Thus, testing therapeutics designed for humans should take this into account. Researchers should consider how the variability of housing density could affect any sex-specific effects they observe by including density as a discovery variable in their analyses.

Many research labs will house rodents individually due to experimental constraints such as needing to record individual drug intake from an animal or caution regarding the risk of damage to surgical implants. However, there is a large field of research examining the negative and sex-specific effects of social isolation housing both in mice and rats [32–34]. For example, while social isolation reduced hippocampal plasticity in both male and female rats, a low dose of ketamine was only able to ameliorate this effect in males [35], suggesting that the antidepressant effects of ketamine depend on context influencing the efficacy of the drug in a sex dependent manner. When studies require single housing of animal models, if a sex-specific effect is found, it is important to investigate whether the same outcome would be seen under different housing conditions. Taken together, in nature, the size of the colony, and in the vivarium, the number of animals in a cage, determines gender-like norms.

### Resource availability

In the wild, rodent roles vary depending on the proximity of a colony to resources such as food or water. For example, in low-resource environments, there is typically one dominant male among a harem of females with the male engaging in defensive territorial behaviors [29, 36]. In higher-resource areas, rodent colonies are more likely to include more than one male with these multiple males coexisting at different social ranks within a dominance hierarchy. Recent research has shown that when a common lab strain of male mice are exposed to a semi natural environment, they too display territorial behavior in low-resource conditions and dominance behaviors in high-resource conditions suggesting this trait has not been changed through domestication [37]. However, the typical cage in the vivarium provides unlimited access to food and water, making it a high-resource environment. Thus, resource abundance shifts the behavioral repertoire of male mice towards dominance behaviors instead of defensive territorial behaviors. Therefore, the physical context changes their role in a sex-specific manner resulting in a gender-like behavior.

Availability of resources also shape female behaviors with female rodents in natural environments typically only displaying territorial aggressive behavior when they are competing for nesting sites for their offspring [38]. In a vivarium environment when a female is pregnant she is isolated and given ample nesting material, therefore the territorial aggressive behaviors shown in wild female mice are not as present in the vivarium environment. Thus, resource availability, an aspect of the physical context, changes behavior in a sex-specific manner producing this gender-like behavior. Moreover, nature and vivarium environments foster two distinct sets of gender-like norms due to their contrasting physical and social contexts. While this section discusses the overall vivarium environment, it is essential to consider the physical context of the cage within the vivarium.

## Physical aspects of cages shape sex-specific roles

The physical attributes of the cage, including the type of cage used and the items placed inside it, may contribute to the development of sex-specific roles within the vivarium environment.

### Cage type effect on rodent gender-like norms

The physical design of the cage is the first important parameter to consider because its structure imposes limits on social interactions. In the last decade, many rodent vivaria have switched to using individually ventilated caging (IVC) systems. While IVC reduces allergen exposure to staff and researchers and limits the spread of infections among the rodents, it creates a significantly different home environment for the animals compared to those housed in conventional filter-top cages [39] or those in nature. For male rats the high rate of air changes in IVC systems, can be aversive [40]. Further, these systems limit the olfactory and auditory cues among cages and thus, could potentially represent a form of social isolation housing [41]. Despite altering olfactory cues, IVC caging does not alter fear conditioning, struggling in the forced swim test, or social exploration [42, 43].

However, IVC systems increase anxiety behavior more in females than males [42, 44]. This sex-specific effect in the lab is especially important because human females are approximately twice as likely to be diagnosed with anxiety disorders as human males [45]. If preclinical models start with a baseline difference in anxiety simply due to housing conditions, it may mask or distort the true mechanistic sex differences we are attempting to identify as contributors to the human clinical findings. Collectively, these studies suggest that cage type is a critical physical context that alters gender-like behaviors. Specific contextual factors of IVC systems—be they differences in olfactory or auditory cues, or air change rates—interact with sex to produce behavioral outcomes distinct from those seen in open-top cages creating gender-like norms. Therefore, understanding the influence of caging parameters like IVC systems on gender-like behavior is crucial for maintaining the internal validity of preclinical research, allowing researchers to identify if observed sex differences in behavior reflect the intended experimental manipulation or unintended influence of the housing environment.

### Enrichment effects on rodent gender-like norms

The addition of accessories to the cage alters the environment and can profoundly affect behavior by creating opportunities or limitations for how sex-specific roles are established. At a minimum, rodents are housed in standard housing conditions with bedding material, a relatively barren environment that significantly limits natural behaviors such as burrowing and nesting. To mitigate this, some guidelines (like those in Europe and Canada) now require basic items such as nesting material or shelter (e.g., igloos, huts, tunnels)—though these standards are not yet mandatory in the United States. Rodents in these standard conditions exhibit elevated anxiety-like, depression-like, stroke, and cardiovascular disease phenotypes compared to rodents in enriched environments, leading to higher morbidity and mortality from stress-sensitive diseases [1]. The consequence is that standard housing conditions induce significant chronic stress in rodents. Critically, this stress creates a unique social context that is well known to affect males and females differently both physiologically and behaviorally [46, 47]. Therefore, stress arising from housing conditions may impact the establishment of gender-like behaviors, potentially biasing anxiety-like or depression-like behaviors assessed in behavioral paradigms.

New developments in caging will also affect gender-like norms in the vivarium. Researchers have shown a growing interest in improving housing conditions through the concept of environmental enrichment. This approach involves introducing running wheels, providing larger cages, or increasing the number of animals in the home cage [48]. Environmental enrichment can reduce anxiety-like and stress responses that improve rodent well-being [49–53]. However, enrichment type and quantity varies widely across labs, potentially contributing to variable experimental results [54]. For example, in a mouse strain known to be aggressive, introducing a running wheel into male group housing increased aggression and disrupted their social hierarchies [17], suggesting the strain and sex of the animal and enrichment type can affect gender-like norms.

Moreover, if enclosures (e.g. igloos, huts, tunnels) are used as enrichment, competition may arise among animals to occupy these spaces, with male mice potentially exhibiting more aggressive forms of competition [55]. Additionally, due to size differences, male mice and rats may find it more challenging to fit within certain enclosures than females. Providing males nesting material in combination with igloos and tunnels has been shown to reduce fighting in mice [56]. Currently, the answer to fighting males is to separate them, however as discussed above, social isolation creates a context that alters gender-like behaviors. Further, subordinate male mice prefer being with their dominant littermate over isolation, suggesting that modifying the cage environment, rather than separation, can reduce gender-like behaviors such as competition [55]. The specific aspects of enrichment that alter sex-specific roles remain understudied, but this knowledge is vital for interpreting the human behavioral relevance of the preclinical model.

Although environmental enrichment improves animal welfare, it can also alter rodent behavior in ways that influence gender-like norms. The key methodological challenge of enrichment is to balance its reduction of chronic stress, anxiety, and morbidity with its alternation of the physical context that will change the expression of gender-like norms in male and female rodents. Consequently, researchers designing preclinical studies must carefully consider the type and amount of enrichment used in order to model gender-like behavior. Failure to account for this *sex X enrichment* interaction can result in behavioral differences (e.g., social dominance or anxiety-like states) that will affect their biology. A first step for taking the environment and its influence on gendered behavior into account is for manuscripts to include a description of the cage environment. Beyond this description, use of cage-tracking software to document how the rodents interact with objects and cage mates would be ideal. Documenting these interactions will help identify factors that change gender-like norms. Exploring these factors can increase the translatability of animal models to the human condition and ensure preclinical research funding supports effective treatments in both female and male humans.

## Social context shapes sex-specific roles

The social context within the cage is profoundly shaped by the presence of multiple rodents. The composition and identity of conspecifics can significantly alter the expression of sex-specific roles. Key factors include the sex composition of the group, the social rank of the individuals, and the familiarity between cage mates.

### Sex composition

Rodents naturally live in mixed sex groups in the wild, but in the vivarium they are separated after weaning and may never interact with the other sex again. While this is done to prevent inbreeding and unwanted litters, it also changes the social context of the animals which in turn changes the roles assigned by sex producing a gender-like norm. In mixed-sex colonies, typically only male rats display aggressive behavior to an intruder, however when the males are removed, females can also show this aggressive behavior [19]. Further, male rats display increased aggressive behavior in a mixed-sex colony compared to an all-male colony, with the presence of females increasing males’ corticosterone level in a low stress environment but decreases it in a high stress environment [57]. Since most rodents in vivaria are housed in same-sex cages post-weaning, this may lead to different gender-like norms than that in natural environments, such as reduced aggression in males and increased aggression in females [19, 57]. Thus, these gender-like norms are malleable when the social context changes much like how human gender norms change as social expectation shifts in humans.

It is not practical to suggest housing rodents in mixed-sex cages. However, the sex composition of the housing room itself can affect performance on operant conditioning tasks, with both male and female rats showing greater accuracy and motivation to complete the task when housed in mixed- compared to same-sex rooms [58]. The factors that contribute to this effect are unknown, but the presence of both sexes may construct a social context that changes the expectations of the rodents in a sex-specific manner contributing to expression of gender-like behavior. Future research could explore this idea by manipulating the sex composition of the housing colony to better understand how this element of the social context can produce behavioral changes that are sex dependent, i.e. how gender-like norms may play a role in their behavior.

Rearing is a critical timepoint when the sex composition of the group profoundly influences the expression of gender-like behaviors. Specifically, the sex composition of both the parents and the offspring significantly alters the expression of parental behavior. In nature, rats and mice often breed in a harem configuration [29, 36]. In contrast, vivarium standard breeding protocols typically involve pair breeding where the male is removed after confirming pregnancy. This standard practice isolates the female rodent throughout pregnancy, birth, and rearing. Consequentially the female is solely responsible for all maternal care and territorial protection, which can increase maternal stress and change their biology in a way that might affect the model for humans.

Conversely, keeping the father in the cage through pregnancy and rearing decreases the rate of infanticide, increases pup growth rate, and results in the male engaging in maternal care behaviors [22]. In addition, keeping multiple females in the same cage through pregnancy and rearing has been shown to decrease anxiety and improve working memory in pups [59], potentially due to more maternal care opportunities for the offspring and decreased stress in the mothers. These findings clearly demonstrate that the parental sex composition is a key component of the social context, which actively shapes and shifts gender-like norms of parental behaviors during the rearing phase.

Furthermore, the sex composition of the litter itself—specifically, the ratio of male to female pups—alters the mother’s care behavior. In humans, mothers often provide more affectionate touch to their sons relative to their daughters [60]. Mother rats, called dams in rodent research, display a similar differential treatment, dedicating more time to licking their male offspring, a critical maternal behavior known to influence brain development [61]. However, studies comparing this behavior between mixed-sex and single-sex litters reveal a complex interaction: dams licked single-sex male litters the least [62]. Correspondingly, male pups raised in single-sex litters exhibit higher anxiety-like behavior compared to males from mixed-sex litters [63]. This demonstrates that social context and experience shape maternal roles. Similarly, if the dam is stressed, female pups experience more adverse treatment than males. This leads to greater negative behavioral outcomes in the female offspring, such as increased expression of depressive-like phenotypes [64]. Ultimately, the sex composition of the litter provides a critical social context that modifies the performance of maternal roles, illustrating that gender-like parenting behavior in rodents is not innate but dynamically adjusted.

The social context is a multi-layered determinant of gender-like norms. It is established not only by the broad sex composition of the colony or housing room (macro-level social context) but also by the immediate sex composition of the parents and the offspring litter (micro-level social context). These factors collectively define the social landscape of the cage, dynamically shaping the expression and expectation of sex-specific roles in rodents leading to the expression of gender-like behaviors. Understanding this hierarchical influence is essential for interpreting behavioral data accurately. Therefore, in order to factor in the effects of the macro and micro level social environment on behavior, the sex composition of the housing room, cage, breeding pairs, and litters should be systematically reported.

### Social rank

The social rank of conspecifics within the cage is another factor that can significantly affect the expression of gender-like norms. The naked mole-rat (NMR) provides a compelling illustration that defined gender-like behaviors shift dramatically based on context which, in the case of the NMR, is their social ranking within the colony [65, 66]. NMR colonies typically consist of one female, the queen, who monopolizes breeding and mates with one to three males. The other colony members effectively become “sexless,” as they are reproductively suppressed and remain prepubescent into adulthood [67]. These non-breeding subordinate NMRs perform foraging, burrow maintenance, and defensive behavior duties [68]. However, if the queen is removed a subordinate NMR can achieve breeding status. This change in social rank is accompanied by changes in sexually differentiated brain regions like the paraventricular region of the hypothalamus [69, 70]. While NMRs have a biological sex from birth, this aspect only becomes behaviorally relevant once they assume a role within their society, i.e., once they “perform” their gender. The NMR illustrates that the social rank of others can alter the behavior of others and influence the expression of gender-like norms.

### Familiarity

Finally, the third component of the social context that alters the expression of gender-like norms is the familiarity of cage mates. Rodents are known to establish territorial boundaries, and this behavior is heavily affected by recognition of another rodent. In the absence of conspecific recognition female rats often express intense territorial aggression with non-familiar conspecifics, yet they can readily cohabitate peacefully in stable social groups when those individuals are familiar [22]. This dynamic demonstrates how the social expectation for aggression—a key gender-like behavior—is contingent upon the familiarity of the cage mate.

In sum, the sex composition, social rank, and familiarity of conspecifics are the key variables that construct the social context within a cage, ultimately shaping the expression and expectation of sex-specific roles forming gender-like norms. Given that different social contexts produce measurable biological ramifications such as changes in neuroanatomy and gene expression, their influence extends beyond behavior to potentially affect response to pharmacological treatments. For example, in male mice, the established social hierarchy can modulate the efficacy of a common selective Serotonin Reuptake Inhibitor, Paroxetine. Specifically, dominant males often show greater improvement in depression-like behaviors, a difference potentially mediated by alterations in monoamine content produced by their social status [71]. Since humans also navigate complex social contexts, treatments developed using simplified models may fail for certain patient populations or, at worst, be detrimental to their health. If unarticulated, these social contexts and the resulting gender-like norms carries significant risks for human translation. Conversely, if these variables are explicitly studied and reported, they can profoundly enhance the applicability of animal models to the human condition. Therefore, the systematic reporting and investigation of these methodological details is not just an opportunity, but a necessity because robust translational outcomes depend on understanding how the social environment shapes the brain and behavior.

## Limitations

For decades, behavioral ecology and genetics have utilized the Gene × Environment (G × E) framework to describe how context modifies physiology and behavior. We acknowledge that our proposed framework of gender-like norms is inherently intertwined with these established concepts. However, research into G × E interactions and sex differences has remained siloed for too long. Introducing the lens of gender-like norms could provide a conceptual bridge to unite these fields. We argue that giving a name to the interactions of biological sex with physical and social context (gender-like norms) adds explanatory value that has been missing in preclinical research. It moves the field beyond viewing sex as a static binary variable and toward an acknowledgement that the factors underlying rodent behavior are as rich and context-dependent as those underlying human behavior.

## Conclusions

Taken together, female and male rodents display unique roles depending on environment in a sex-specific manner that, here, we call gender-like behavior. This is seen clearly when comparing the natural environment to the vivarium environment, as these two spheres construct distinct physical and social contexts that, in turn, produce their own set of gender-like norms. Furthermore, exploring the physical and social contexts *within the cage* demonstrates how often-overlooked and underreported factors—such as cage type, enrichment, and the presence of others—significantly contribute to the construction of gender-like norms in preclinical rodent models. Whether it is a non-reproducing female naked mole rat becoming a queen when the right conditions are met, or a female rat becoming aggressive when males are absent, in many of the examples provided, rodents are actively and collaboratively performing their roles in a social environment. According to gender theorists, gender is not a fixed identity but rather a performance shaped by ongoing behavior and social expectations [18]. Thus, performance of context dependent behaviors is the foundation of gender theory [72, 73]. Taking this perspective, we see how nature as well as the vivarium environment, set the stage for gender performance in rodents.

We believe that this is a critical framework missing from current rodent models of human conditions. Adding this perspective will make our research more rigorous, reproducible, and applicable to the human condition. While all the rodent gender-like behaviors we outlined may appear to only increase the complexity of experiments, in order for there to be a real translatability to the human condition, maybe it’s time to try embracing complexity.

## Data Availability

No datasets were generated or analysed during the current study.

## Abbreviations

IVC: Individually ventilated caging
NMR: Naked mole rat
